# Surgical Correction of Large Talar Tilt in Varus Ankle Osteoarthritis II: A New Treatment Algorithm Based on the Tibial Plafond Inclination and Arthritis Types

**DOI:** 10.3390/jcm15041580

**Published:** 2026-02-17

**Authors:** Jun Young Choi, Jin Soo Suh

**Affiliations:** Department of Orthopedic Surgery, Inje University Ilsan Paik Hospital, 170 Juhwa-ro, Ilsanseo-gu, Goyang 10380, Republic of Korea; sjs0506@paik.ac.kr

**Keywords:** ankle, osteoarthritis, varus talar tilt, joint-preserving surgery, fibular osteotomy, inframalleolar correction, supramalleolar osteotomy, algorithm

## Abstract

Medial opening-wedge supramalleolar osteotomy (MOWSMO) is a joint-preserving surgical option for varus ankle osteoarthritis (OA); however, its ability to correct large varus talar tilt (TT), particularly in advanced diseases like Takakura stage IIIB, remains limited. Varus TT represents a complex three-dimensional deformity characterized by coronal malalignment and internal rotation, which cannot be reliably corrected by isolated supramalleolar realignment. Building on our previous work, we propose a new treatment algorithm for large varus TT based on preoperative tibial plafond inclination (TPI) and arthritis type, categorized as translational and rotational. While MOWSMO primarily addresses TPI, effective correction of talar inclination requires a balanced, multilevel approach. This includes using an oblique sliding fibular osteotomy to facilitate rotational realignment with fibular shortening and, critically, prioritizing inframalleolar correction (IMC). IMC is implemented through an “all-in-one” strategy involving lateral ligament repair, deltoid ligament release, calcaneal osteotomy, and posterior tibial tendon lengthening. Furthermore, we discuss critical intraoperative considerations, such as avoiding excessive TPI valgization to prevent a “paradoxical increase” in TT. Collectively, this framework provides clinically relevant insights and a reproducible algorithm for achieving satisfactory outcomes in the joint-preserving management of severe varus ankle OA.

## 1. Introduction

Varus ankle osteoarthritis (OA) can arise from post-traumatic changes; however, it also clearly exists as a degenerative condition. Although the exact incidence of degenerative varus ankle OA remains uncertain, a disproportionate number of related studies have been reported in East Asia compared with Western populations, suggesting potential ethnic or regional differences in prevalence [1,2]. Takakura et al. [1] and Tanaka et al. [3,4] classified varus ankle OA into four stages: Stage I, characterized by early degenerative changes with subchondral sclerosis and minimal joint-space narrowing; Stage II, defined by medial joint-space narrowing without complete obliteration; Stage IIIA, in which joint-space obliteration with subchondral bone contact occurs between the medial talar gutter and the medial malleolus; Stage IIIB, representing further progression of joint space obliteration extending to the roof of the talar dome with subchondral bone contact; Stage IV, marked by complete joint destruction involving both the medial and lateral compartments.

According to this classification, progression from Stage IIIA to Stage IIIB is accompanied by erosion of the medial malleolus and a progressive increase in varus talar tilt (TT), ultimately resulting in direct subchondral bone contact between the medial talar dome and the tibial plafond. Medial opening-wedge supramalleolar osteotomy (MOWSMO), originally introduced by Takakura et al. [1], is an effective joint-preserving procedure for patients with Stage II and Stage IIIA varus ankle OA [5,6,7,8,9,10]. However, for Stage IIIB disease, consensus regarding an effective corrective strategy remains lacking. We believe that this therapeutic gap largely reflects differences between the correction of varus TT and that of varus knee OA. In contrast to the knee, isolated coronal-plane realignment of the distal tibia is insufficient in most cases for two key reasons. First, the fibula plays a critical role in determining talar position through its buttressing effect and contribution to distal tibiofibular stability [11,12,13,14,15]. Second, varus TT represents a complex three-dimensional deformity involving not only coronal malalignment but also rotational and translational components arising from the intricate biomechanical interactions between the distal tibiofibular complex and tarsal bones [16,17].

In our previous article [18], we highlighted these challenges and summarized the currently available evidence. Building on this work, we propose a novel treatment algorithm specifically designed to correct varus TT and demonstrate its application in clinical practice.

In our previous article [18], we highlighted these challenges and summarized the currently available evidence. Building on this work, we propose a novel treatment algorithm specifically designed to correct varus TT and demonstrate its application in clinical practice. Our discussion begins by illustrating how varus TT can manifest under various conditions, specifically focusing on the differentiation between rotational and translational types and the influence of tibial plafond inclination (TPI). Furthermore, we present detailed clinical cases to illustrate the practical implementation of our surgical strategies, providing a comprehensive guide for managing varus ankle OA with large TT.

## 2. Key Components of Surgical Treatment Before Applying the New Algorithm

### 2.1. Fundamental Radiographic Parameters

**Figure 1:**
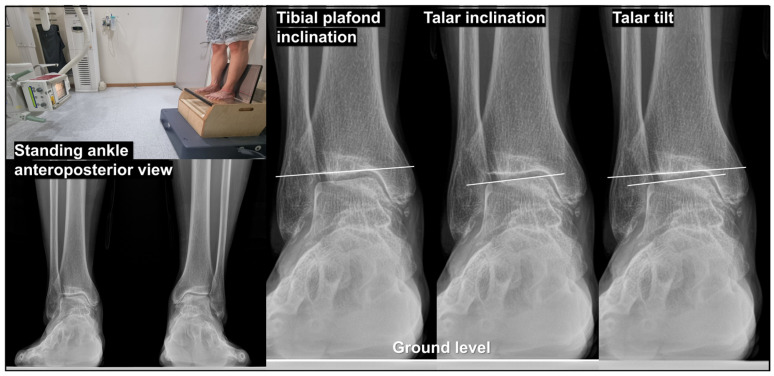
Measurement of fundamental radiographic parameters on the standing ankle anteroposterior (AP) view. The standing ankle AP radiograph is obtained under full weight-bearing conditions with both ankles included on a single cassette. Tibial plafond inclination (TPI) is defined as the angle between the tibial plafond and the ground level. Talar inclination (TI) is defined as the angle between the talar dome and the ground. Talar tilt (TT) is calculated by subtracting TPI from TI.

To accurately characterize the pattern of varus TT, all radiographic examinations should be obtained under weight-bearing conditions. In our practice, the standing ankle anteroposterior (AP) view is acquired with the patient standing comfortably, with both ankles included on a single cassette and positioned approximately shoulder-width apart (Figure 1). In addition, standing ankle lateral, hip-to-talus [19], hip-to-calcaneus [20], and hindfoot alignment views [21,22] are routinely obtained as part of the baseline radiographic assessment. Valgus stress radiographs are obtained to evaluate the flexibility of the varus TT. A standardized load of 150 N is applied to the ankle joint using a Telos device (METAX, Hungen, Germany). In most cases, when TT is correctable on valgus stress views, a comparable degree of correction can be achieved through joint-preserving surgical procedures. Correction exceeding this degree observed on valgus stress radiographs is rare.

On standing ankle AP radiographs (Figure 1), measurement of TPI, talar inclination (TI), and TT is essential. TPI is defined as the angle between the tibial plafond and the ground, with values between 4° of valgus and 4° of varus considered within a neutral range. TPI generally reflects proximal lower-limb alignment: in the absence of tibial shaft trauma or deformity, knee valgus malalignment is typically associated with valgus TPI, whereas varus knee malalignment corresponds to varus TPI. TI is defined as the angle between the talus and the ground and is typically varus in ankle OAs with varus TT. TT is calculated by subtracting TPI from TI.

### 2.2. Two Different Types of Varus Ankle Osteoarthritis

Varus ankle OA can present with distinct pathomechanical patterns with important implications for surgical planning [18,23]. One such pattern is the rotational type, most commonly associated with pes cavovarus deformity. In this type, progressive varus talar orientation leads to subchondral bone contact between the medial talar dome and the tibial plafond. Despite substantial varus TT, the talus remains relatively centered within the ankle mortise, with minimal medial translation. In rotational-type varus ankle OA, correction with MOWSMO is frequently insufficient, and successful treatment requires addressing the underlying pes cavovarus deformity. Furthermore, identifying the etiology of pes cavovarus is essential, and the presence of a paralytic component should be evaluated preoperatively using electromyography and nerve conduction velocity studies.

**Figure 2:**
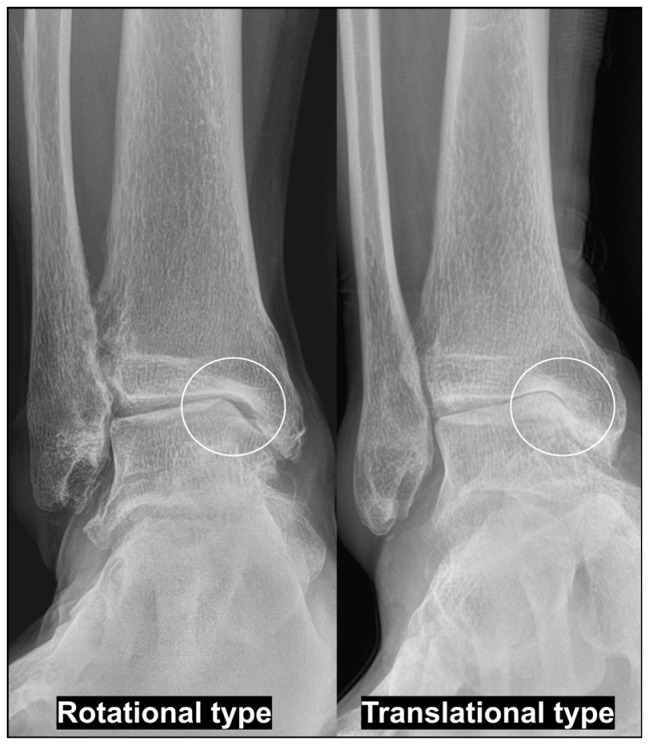
Two types of varus ankle osteoarthritis. Careful assessment of the obliteration of the space between the medial malleolus and medial talar gutter is essential; obliteration is typically absent in the rotational type, whereas it is present in the translational type.

In contrast, the translational-type varus ankle OA refers to a progressively developing varus TT that occurs during the natural course of degenerative varus ankle OA. This pattern is characterized by a gradual medial talar translation within the ankle joint, accompanied by an increase in varus TT and medial malleolar erosion (Figure 2). In these cases, MOWSMO effectively facilitates lateralization of the talus, and varus TT can often be successfully corrected with MOWSMO combined with additional joint-preserving procedures.

### 2.3. Role and Limitations of Supramalleolar Osteotomy in Correcting Varus Talar Tilt

Several joint-preserving surgical options for SMO are available for ankle OA accompanied by varus TT, including lateral closing-wedge SMO [24], dome osteotomy [25,26], and MOWSMO. Dome osteotomy is an important treatment option; however, its indication for correcting varus TT differs from that of MOWSMO, as discussed later.

MOWSMO has been extensively studied and can reliably induce lateral translation of the talus; however, the amount of varus TT correction achievable with MOWSMO is generally limited to approximately 2–3° [27]. Accordingly, favorable outcomes are expected in Takakura stage II or IIIA ankle OA, whereas results in Takakura stage IIIB become increasingly unpredictable as TT severity increases. Based on our clinical experience and consistent with Tanaka et al. [4], patients with Takakura stage IIIB ankle OA with a TT < 10° can achieve satisfactory outcomes with MOWSMO combined with fibular osteotomy. However, when TT exceeds 10°, supramalleolar correction alone is unlikely to be sufficient, and additional procedures, such as inframalleolar correction (IMC), are typically required. The following surgical tips are essential when performing MOWSMO for varus TT correction.

**Figure 3:**
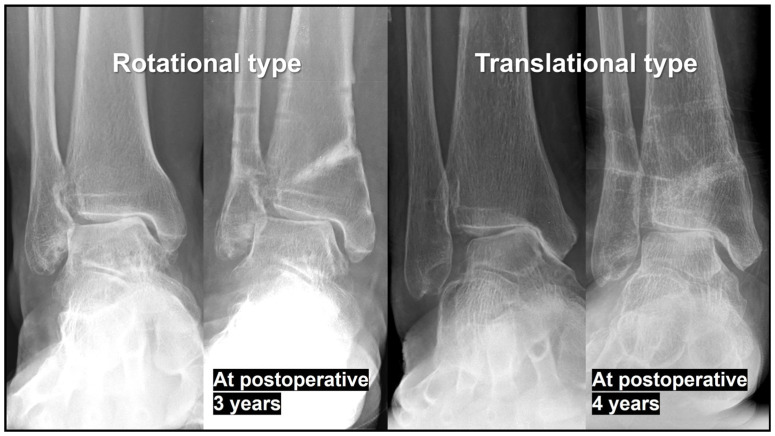
Paradoxical increase in talar tilt (TT) following excessive of tibial plafond inclination (TPI) valgization. Excessive TPI valgization resulted in a paradoxical increase in TT, leading to an increased medial joint pressure. Over time, this abnormal load distribution accelerated the progression of medial ankle osteoarthritis, ultimately necessitating revisional, joint-sacrificing surgery at final follow-up. As the condition advanced and osteoarthritis extended to the lateral side, the TT subsequently appeared to decrease, as seen in the translational type example. This mechanism is relevant to both rotational and translational types of varus ankle osteoarthritis.

1. Lowering the tibial osteotomy line to the distal third of the syndesmosis, rather than the proximal third as in conventional SMO, is advantageous [28].
2. Approximately 1 mm of medial opening corresponds to approximately 1° of TPI valgization.
3. During osteotomy site opening, hinge fracture and unintended distraction of the osteotomy site must be meticulously avoided; controlled opening around an intact hinge is critical [29,30].
4. When correcting TPI, excessive valgization should be avoided; TPI should not exceed 4° of valgus. Ideally, the tibial plafond should be restored to a position parallel to the ground. In our experience, TI correction is usually limited to achieving a position parallel to the ground, regardless of the surgical technique, and conversion to a valgus position relative to the ground is rarely possible. If TI remains in varus, overcorrection of TPI may paradoxically increase TT, a phenomenon we refer to as ‘paradoxical TT increase’ (Figure 3). This increase may accelerate the OA progression and ultimately necessitate revisional, joint-sacrificing surgery.

In summary, because excessive TPI correction is neither required nor desirable during MOWSMO for varus TT, a medial opening of >5 mm is rarely necessary. Avoiding TPI overcorrection is the most critical consideration in this procedure, particularly in patients with Takakura stage IIB OA.

### 2.4. Role of Fibular Osteotomy and the Authors’ Preferred Technique

Fibular osteotomy performed in conjunction with MOWSMO plays a significant role in restoring ankle alignment [11,31,32,33,34]. Biomechanically, the fibula contributes not only to lateral ankle stability but also to axial load transmission across the ankle joint [12,13,14,15,35]. Loss of this modest yet essential weight-bearing function increases load concentration on the lateral tibial plafond and compromises the buttressing effect of the lateral malleolus on the talus. Clinical observations in patients with fibular fracture malunion further support the fibula’s influence on talar alignment, as posttraumatic valgus TT frequently develops. These findings imply that fibular osteotomy, even when performed independently of SMO, can induce valgus reorientation of the talus. To achieve this effect predictably, intentional shortening, external rotation, and valgization of the distal fibular fragment are critical procedural components [36].

**Figure 4:**
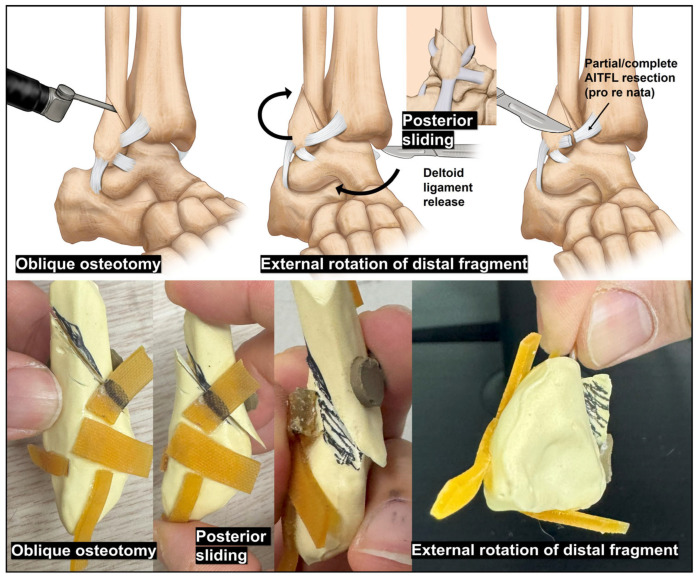
Illustration and sawbone model of oblique sliding fibular osteotomy. After performing an oblique osteotomy of the lateral malleolus with the release of the deltoid ligament, valgus and external rotation forces were applied to the ankle by an assistant. At the same time, talar alignment was continuously assessed under fluoroscopic guidance, with the goal of restoring a neutral talar tilt. As reduction progressed, the distal fibular fragment generally migrated in a posterosuperior direction and demonstrated a mild degree of external rotation. If this maneuver failed to provide sufficient repositioning of the distal fibular fragment, partial or complete resection of the anteroinferior tibiofibular ligament at the fibular insertion was selectively performed. The Sawbone model experiments further illustrated that the distal fibular fragment rotated externally when viewed from an inferior perspective. *AITFL, anteroinferior tibiofibular ligament*.

We currently favor an oblique sliding fibular osteotomy (OSFO; Figure 4), inspired by the fracture morphology observed in supination–external rotation–type ankle injuries [36]. The osteotomy begins near the attachment of the anteroinferior tibiofibular ligament (AITFL) anteriorly and extends posterosuperiorly. Varus TT is often accompanied by internal talar rotation [16]; therefore, when the anterior talofibular ligament (ATFL) is intact, an osteotomy that facilitates external rotation of the distal fibular fragment may theoretically promote corresponding external rotation of the talus. This rotational correction mechanism is a key conceptual advantage of the OSFO.

In practice, fixation of the fibular osteotomy is performed after all other bony alignment corrections. By externally rotating and valgizing the ankle at this stage, the distal fibular fragment naturally translates posteriorly and rotates externally along the oblique osteotomy plane. When necessary, a partial or total cut of the AITFL may be performed to optimize fragment positioning [37]. Fixation using two or three 1.4- or 1.6 mm Kirschner wires across the osteotomy site provides stable maintenance of the achieved correction and is considered the preferred strategy in our experience.

### 2.5. Importance of Inframalleolar Correction

Since Lee et al. [38] reported the use of calcaneal osteotomy combined with lateral ligament repair as a joint-preserving surgical option for varus ankle OA, IMC has received comparatively limited attention, with most studies focusing on supramalleolar correction. Previous studies evaluating the effect of adding IMC to MOWSMO have introduced various adjunctive procedures [27,39,40,41,42] and have consistently concluded that the addition of IMC to MOWSMO facilitates the correction of varus TT.

**Figure 5:**
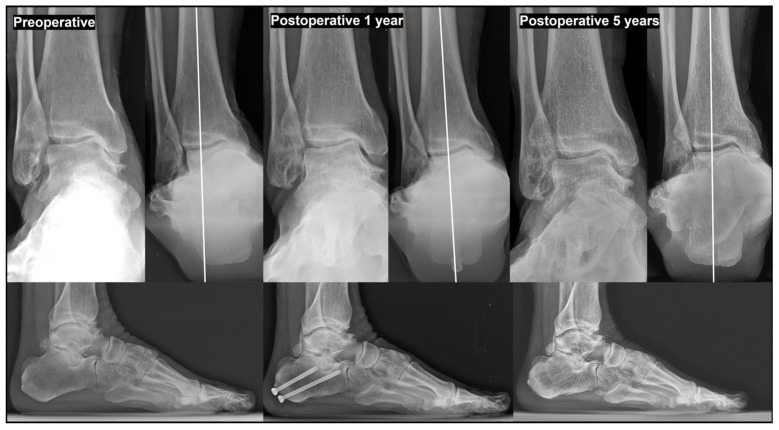
Effect of four combined procedures to achieve inframalleolar correction for advanced varus ankle osteoarthritis. A 62-year-old male patient with Takakura stage IIIB varus ankle osteoarthritis treated with inframalleolar correction, including anterior talofibular ligament repair, deltoid ligament release, lateral closing wedge calcaneal osteotomy with lateral displacement, and posterior tibial tendon lengthening without supramalleolar or fibular osteotomy. At 1-year postoperatively, the varus talar tilt was corrected with effective widening of the medial clear space, and radiographic correction was well maintained without deterioration at the 5-year follow-up.

Likewise, based on our clinical experience, the role of IMC in correcting varus TT deserves substantially greater emphasis. IMC should not be regarded merely as an ancillary procedure for MOWSMO. In selected cases, IMC can be performed as a standalone procedure without MOWSMO and fibular osteotomy (Figure 5) or in combination with fibular osteotomy without MOWSMO.

From an anatomical and biomechanical perspective, the key components involved in IMC include the ATFL, deltoid ligament, calcaneus (or subtalar joint), and posterior tibial tendon (PTT). In most cases, we adopt an “all-in-one” surgical strategy combining ATFL repair or reconstruction, deltoid ligament release, calcaneal osteotomy, and PTT lengthening [43]. Several technical considerations are critical when performing combined IMC procedures.

1. Before ATFL repair or reconstruction, meticulous removal of large bony spurs at the lateral malleolus is essential because prominent osteophyte formation is common in varus ankle OA [44]. When fibular osteotomy is performed concurrently, ATFL repair becomes technically easier after fixation of the fibular osteotomy.
2. Deltoid ligament release is performed at the medial malleolar attachment site with complete detachment of both the superficial and deep components.
3. Calcaneal osteotomy is performed as a lateral closing wedge osteotomy of approximately 1 cm [45]. After removing the wedge fragment, the posterior calcaneal fragment should be displaced laterally as much as possible, followed by fixation with two 5.0 or 6.5 mm cannulated screws placed across the osteotomy site.
4. PTT lengthening is performed preferentially distal to the tarsal tunnel. Considering the excursion capacity of PTT [46], a lengthening of approximately 2 cm is generally sufficient.

### 2.6. Management of Exceptionally Large Varus Talar Tilt

Based on our clinical experience, joint-preserving surgery is generally recommended for varus TT up to approximately 15°. Such procedures are contraindicated when TT exceeds 20°. A varus TT > 20°, particularly of the rotational type, is often associated with paralytic pes cavovarus deformities. In these cases, the pathological condition fundamentally differs from that of the patient population included in the present study, and a distinct treatment strategy is required.

**Figure 6:**
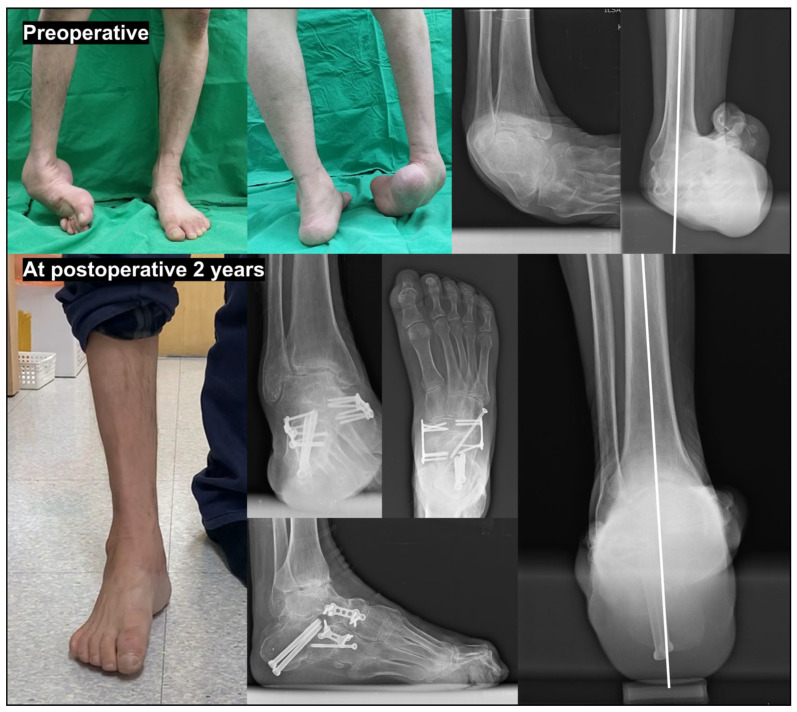
Surgical correction of severe pes cavovarus deformity secondary to post-polio syndrome. A 57-year-old man presented with severe pes cavovarus deformity secondary to post-polio syndrome. Preoperative clinical photographs and radiographs revealed a rigid cavovarus deformity with hindfoot varus, forefoot-driven cavus, and equinus contracture. A combination of procedures was performed to restore the plantigrade foot, including open Achilles tendon lengthening, plantar fasciotomy, Dwyer’s calcaneal osteotomy with talonavicular and calcaneocuboid joint arthrodesis, and posterior tibial tendon transfer to the lateral cuneiform. At 2 years postoperatively, clinical photographs and radiographs demonstrate successful correction of the cavovarus deformity, restoration of a plantigrade foot, and satisfactory overall alignment of the hindfoot and midfoot.

Depending on the location and severity of the pes cavovarus deformity, adjunctive procedures may be required to address associated hindfoot and forefoot components. These include first metatarsal closing-wedge osteotomy, peroneus longus–to–brevis or PTT transfer, Achilles tendon lengthening, and calcaneal osteotomy. In rigid or fixed pes cavovarus deformities, more extensive corrective procedures, such as double or triple arthrodesis, may be necessary (Figure 6).

Similarly, joint-preserving surgery is not recommended for translational-type varus ankle OA with a TT of >20°. Instead, ankle arthrodesis or selective total ankle replacement arthroplasty should be considered, depending on patient-specific factors.

## 3. A New Treatment Algorithm Based on the Tibial Plafond Inclination and Arthritis Types

**Figure 7:**
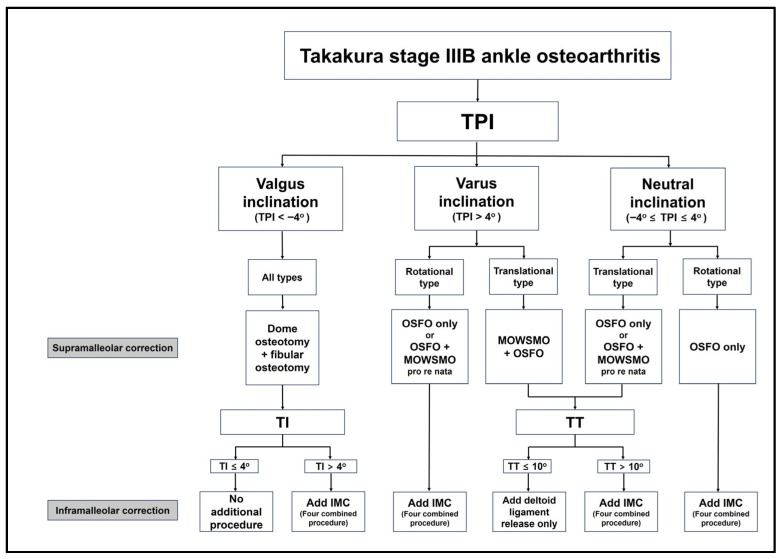
A new treatment algorithm for joint-preserving surgery for patients with Takakura stage IIIB ankle osteoarthritis based on the preoperative tibial plafond inclination and arthritis type. All numerical values are reported using a sign convention in which valgus alignment is assigned negative values, and varus alignment positive values. *TPI, tibial plafond inclination; OSFO, oblique sliding fibular osteotomy; MOWSMO, medial opening wedge supramalleolar osteotomy; TI, talar inclination; TT, talar tilt; IMC, inframalleolar correction*.

Building on the key components discussed above, a new treatment algorithm based on preoperative TPI and arthritis type is presented in Figure 7. We propose that correction of varus TT should be approached from two complementary perspectives: correction of TPI and correction of TI. TPI correction can be achieved through SMO, whereas TI correction can be accomplished through fibular osteotomy and IMC.

### 3.1. Correction of Varus Ankle Osteoarthritis with Valgus Tibial Plafond Inclination

**Figure 8:**
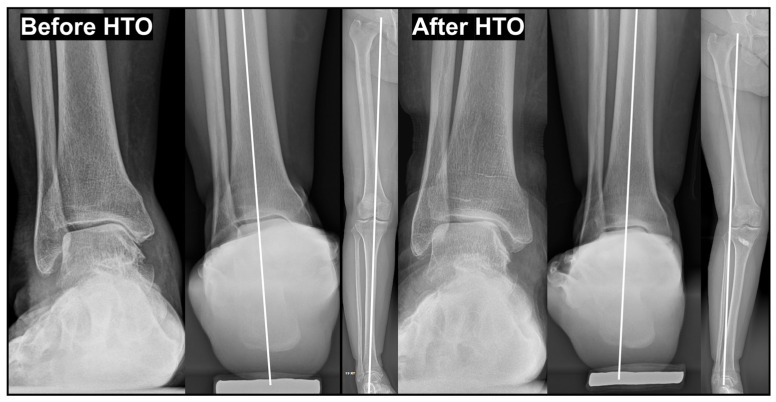
Development of rotational type varus ankle osteoarthritis following knee valgization after medial opening wedge high tibial osteotomy (HTO). Preoperative and postoperative standing radiographs demonstrate valgization of the knee joint following HTO. The resultant proximal realignment led to a concomitant change in the tibial plafond inclination toward valgus. Despite correction at the knee joint level, this alteration in distal tibial orientation resulted in relative varus alignment at the ankle, contributing to the development of varus ankle osteoarthritis. *HTO, medial opening wedge high tibial osteotomy*.

Varus ankle OA accompanied by valgus TPI is frequently associated with an abrupt change in knee joint alignment from varus to valgus. Such changes are commonly observed after procedures such as medial opening-wedge high tibial osteotomy (Figure 8) or total knee replacement arthroplasty. In this setting, the talus is typically oriented nearly parallel to the ground or shows only a mild varus tilt. Because the varus TT in these cases is more likely related to a sudden alteration in proximal alignment rather than a slowly progressive degenerative process, a rotational-type pattern is often observed.

For the correction of valgus TPI, a medial closing-wedge SMO may be considered. However, dome osteotomy combined with fibular osteotomy, as described by Kim et al. [25], may be more suitable for patients with opposing coronal plane deformities between the ankle and lower limbs. In their study, dome SMO effectively corrects the local ankle deformity while simultaneously restoring the hip–knee–ankle axis toward neutral alignment. Additionally, the redistribution of joint loading toward the relatively preserved areas of the ankle joint was observed.

In patients with varus ankle OA accompanied by valgus TPI, the primary technical objective during dome osteotomy is to restore the tibial plafond to a position as parallel to the ground as possible. Regarding IMC, it may not be required when the preoperative TI is ≤4° (i.e., the talus remains nearly parallel to the ground). In contrast, if TI exceeds 4° varus, adjunctive IMC should be considered to achieve sufficient correction and optimize overall talar realignment.

### 3.2. Correction of Varus Ankle Osteoarthritis with Varus Tibial Plafond Inclination

In varus ankle OA with varus TPI, the underlying cause may originate from two different mechanisms: proximal varus malalignment, most commonly related to knee joint alignment, or intrinsic varus orientation of the tibial plafond relative to the tibial shaft.

**Figure 9:**
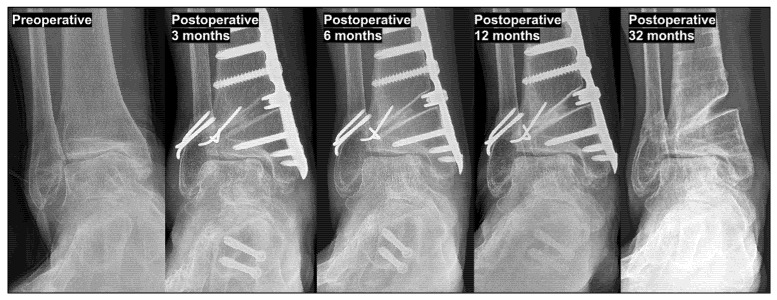
A paradoxical increase after excessive tibial plafond valgization. In rotational-type varus ankle osteoarthritis with varus tibial plafond inclination (TPI, 8°), excessive valgization of the TPI results in a paradoxical increase in talar tilt (TT) during the early postoperative period, leading to heightened medial joint pressure. Notably, correction of the talar inclination can be achieved up to a neutral level. Over time, this abnormal load distribution accelerates the progression of medial compartment osteoarthritis (OA). As the condition advances toward global OA, TT subsequently appears to decrease due to comprehensive joint destruction and structural remodeling.

**Figure 10:**
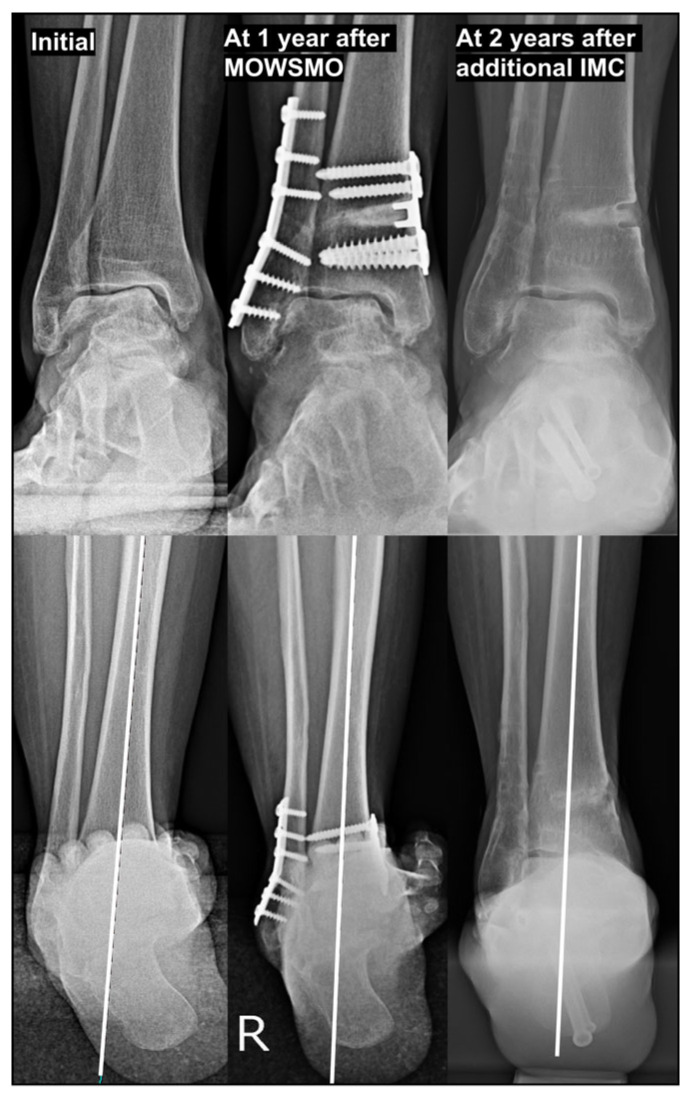
The necessity of inframalleolar correction in rotational-type varus ankle osteoarthritis. The initial radiographs of a 57-year-old woman with rotational-type varus ankle osteoarthritis demonstrated varus talar tilt (TT) without medial talar translation. Supramalleolar correction using medial opening-wedge supramalleolar osteotomy combined with fibular osteotomy was performed as the first-stage procedure. However, at the 1-year follow-up, correction of the TT was limited to approximately 2° with persistent ankle pain. Because of ongoing symptoms and insufficient correction, the hardware was removed 1.5 years postoperatively, and IMC using four combined procedures was simultaneously performed. Two years after the second surgery, radiographs demonstrated dramatic improvement in TT and hindfoot alignment, accompanied by marked clinical symptom relief. *MOWSMO, medial opening wedge supramalleolar osteotomy; IMC, inframalleolar correction*.

Lateral translation of the talus is not required in rotational-type varus ankle OA with varus TPI. Therefore, MOWSMO generally provides limited benefit in this setting and may even induce a paradoxical increase in TT (Figure 9); nonetheless, for patients with a substantial varus TPI (greater than 10°), MOWSMO remains a viable surgical option to address the underlying bony malalignment. In contrast, OSFO should be performed at a minimum to facilitate talar valgization, whereas IMC serves as the primary procedure for deformity correction (Figure 10).

**Figure 11:**
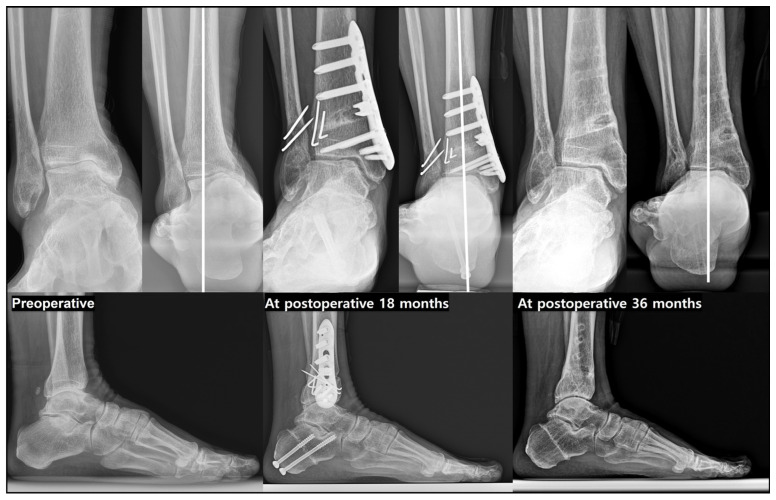
The ‘All-in-one’ procedure performed in a patient with translational-type varus ankle osteoarthritis with varus tibial plafond inclination. Preoperative radiographs demonstrate translational-type varus ankle osteoarthritis with varus tibial plafond inclination, characterized by pronounced varus talar tilt (TT) and medial talar translation. Medial opening wedge supramalleolar osteotomy, oblique sliding fibular osteotomy, and inframalleolar correction were performed in combination. At 18 and 36 months postoperatively, serial radiographs show marked correction of the TT and successful lateral talar translation, with restoration of ankle alignment and maintenance of correction over time.

In the translational type, both the lateral talar translation and varus TPI correction are required. Because these goals can be achieved simultaneously, MOWSMO combined with OSFO is the preferred surgical option. Even in this setting, the target TPI should be restored to a neutral position (parallel to the ground), and excessive valgization should be avoided. When preoperative TT is ≤10°, satisfactory outcomes can often be achieved with additional deltoid ligament release alongside OSFO and MOWSMO. However, when TT exceeds 10° preoperatively, adjunctive IMC should be strongly considered (Figure 11).

### 3.3. Correction of Varus Ankle Osteoarthritis with Neutral Tibial Plfond Inclination

Clinically, varus ankle OA with neutral TPI is the most common presentation. In rotational-type varus OA with neutral TPI, TPI correction is unnecessary, and lateral talar translation is not required. Therefore, deformity correction using OSFO combined with IMC is recommended.

For translational-type varus OA with a neutral TPI, MOWSMO combined with OSFO may be used to address medial talar translation. In this situation, it is critical to limit the medial opening to <5 mm to avoid overcorrection of the TPI. The need for additional IMC should be determined based on the preoperative TT; specifically, if TT exceeds 10°, the adjunctive IMC should be considered to achieve an adequate correction.

**Figure 12:**
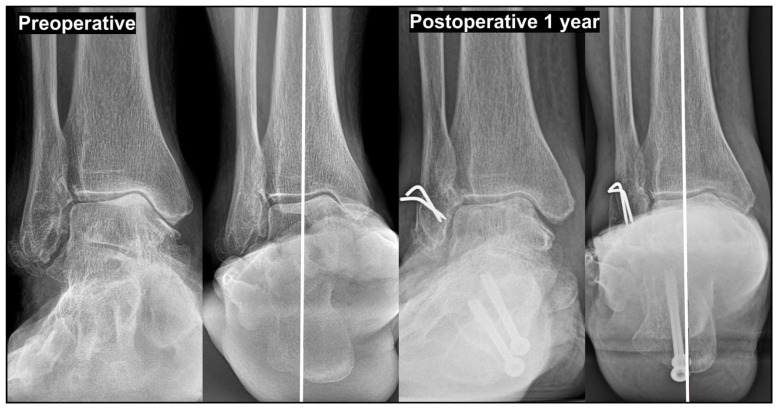
Correction of translational-type varus ankle osteoarthritis with a neutral tibial plafond inclination without supramalleolar osteotomy. Preoperative radiographs demonstrate translational-type varus ankle osteoarthritis with neutral tibial plafond inclination, characterized by varus talar tilt and medial translation of the talus. An oblique sliding fibular osteotomy with inframalleolar correction was performed without supramalleolar osteotomy. At 1 year postoperatively, radiographs show successful correction of the talar tilt and lateral talar translation, with pain relief.

To reduce the risk of excessive valgization of the tibial plafond, an alternative strategy—analogous to that applied in rotational-type deformities—can be used in translational-type cases, where deformity correction is achieved predominantly through OSFO and IMC (Figure 12).

## 4. Postoperative Management

Following surgery, a below-knee splint was initially applied. Approximately one week later, once wound healing was confirmed, the splint was transitioned to a below-knee cast. During the casting process, we intentionally immobilized the ankle in a position of maximal valgus and external rotation. To ensure the talus remained in the desired neutral alignment, fluoroscopic imaging was routinely utilized during application. This specific positioning is designed to provide adequate distraction between the medial talar dome and the tibial plafond, facilitating the development of fibrocartilage in the medial joint space during the early healing phase [18].

A 6-week period of strict non-weight-bearing was mandatory while in the cast, and thorough patient counseling was conducted to ensure compliance. After the cast was removed at the 6-week mark, patients transitioned to a walking boot, starting with partial weight-bearing supported by a single crutch. Progression to full weight-bearing without any orthotic assistance was permitted at 3 months postoperatively, followed by a stepwise return to athletic activities.

## 5. Conclusions

Based on our experience with joint-preserving procedures for varus TT, several clinically relevant insights emerge. First, IMC plays a more critical role than SMO in correcting TT. The corrective effect of MOWSMO on TT is limited, generally not exceeding 2–3°, indicating that reliance on MOWSMO alone is insufficient in most cases.

Second, when greater TT correction is required, adjunctive procedures such as fibular shortening and external rotation should be considered, as they substantially enhance IMC. Third, complete talar valgization relative to the ground is rarely achievable even with combined corrective procedures. In practice, the realistic surgical goal is to restore the talus to a position parallel to the ground. Finally, during MOWSMO, particular attention should be paid to restoring the tibial plafond parallel to the ground, as overcorrection can paradoxically increase TT. Collectively, these findings underscore the importance of prioritizing IMC and adopting a balanced multilevel approach when managing varus TT in joint-preserving ankle surgery.

In conclusion, this study introduces a comprehensive treatment algorithm tailored to the specific morphology of varus TT—specifically distinguishing between rotational and translational types and utilizing the TPI as a key decision-making criterion. While the proposed algorithm is not yet definitive and challenges remain in correcting all cases of large varus TT, our clinical experience demonstrates that it provides a logical and reproducible framework for achieving satisfactory outcomes. By progressively addressing existing knowledge gaps, this algorithm serves as a practical guide for surgeons navigating the complexities of joint-preserving ankle surgery. Ongoing and future studies will further refine and validate this approach, ultimately leading to a more reliable and standardized management strategy for varus ankle OA with large TT.

## Data Availability

The data that support the findings of this study are available from the corresponding author [J.Y.C.], upon reasonable request.

## Abbreviations

The following abbreviations are used in this manuscript:

OA	Osteoarthritis
TT	Talar Tilt
TPI	Tibial Plafond Inclination
TI	Talar Inclination
MOWSMO	Medial Opening-Wedge Supramalleolar Osteotomy
IMC	Inframalleolar Correction
ATFL	Anterior Talofibular Ligament
AITFL	Anteoinferior Talofibular Ligament
PTT	Posterior Tibial Tendon
SMO	Supramalleolar Osteotomy
OSFO	Oblique Sliding Fibular Osteotomy
AP	Anteroposterior
HTO	Medial Opening Wedge High Tibial Osteotomy
